# Anastomotic stenosis of bioengineered trachea grafts is driven by transforming growth factor β1-induced signaling, proinflammatory macrophages, and delayed epithelialization

**DOI:** 10.1016/j.xjon.2023.07.016

**Published:** 2023-07-27

**Authors:** Joanna Weber, Russell Seth Martins, Zaid Muslim, Mirza Zain Baig, Kostantinos Poulikidis, Al Haitham Al Shetawi, Faiz Y. Bhora

**Affiliations:** a Division of Thoracic Surgery, Department of Surgery, Hackensack Meridian School of Medicine, Hackensack Meridian Health Network, Edison, NJ; b Department of Surgery, Cleveland Clinic, Cleveland, Ohio; c Department of Surgery, Howard University, Washington, DC; d Divisions of Surgical Oncology and Oral & Maxillofacial Surgery, Department of Surgery, Vassar Brothers Medical Center, Nuvance Health, Dyson Center for Cancer Care, Poughkeepsie, NY

**Keywords:** tissue engineering, trachea, granulation tissue, stenosis, inflammation

## Abstract

**Objective:**

Anastomotic stenosis caused by hypertrophic granulation tissue often develops in response to orthotopically implanted bioengineered tracheal grafts. To determine mechanisms responsible for the development and persistence of this granulation tissue, we looked for changes in gene expression from tissue specimens from the graft-native interface.

**Methods:**

RNA was isolated from paraffin-embedded tissue samples of the anastomotic sites of orthotopically implanted bioengineered tracheal grafts of 9 animals. Tissue samples were binned into 3 groups based on degree of stenosis: no stenosis (<5%), mild stenosis (25%-50%), and moderate and severe stenosis (≥75%). Sections of healthy trachea tissue were used as control. The expression levels of ∼200 genes related to wound healing, plus several endogenous controls, were measured with a pathway-focused predesigned primer array.

**Results:**

Expression of ARG2, IL4, RPL13 A, TGFBR3, and EGFR decreased, whereas expression of RUNX2 was increased in stenotic wounds compared with nonstenotic tissue. Based on the cell types present in the trachea and wound healing, this expression profile indicates a lack of M2 anti-inflammatory macrophages, absent epithelial cells, and transforming growth factor β1-induced signaling.

**Conclusions:**

These findings represent a significant step for tracheal tissue engineering by identifying several key mechanisms present in stenotic granulation tissue. Further research must be conducted to determine what modifications of the graft substrate and which coadministered therapeutics can be used to prevent the development of hypertrophic granulation tissue.

**Figure:**
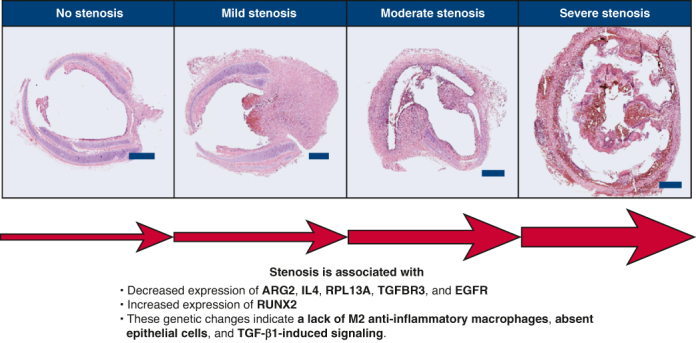
Gene expression in anastomotic stenosis of implanted bioengineered trachea grafts.

**Central Message**

Gene expression in granulation tissue from stenosed bioengineered tracheal graft implants reveals TGF-β1–induced signaling, delayed re-epithelialization, and persisting proinflammatory macrophages.

**Perspective**

Although anastomotic stenosis and hypertrophic granulation tissue is a common cause of failure across existing tracheal graft designs, few studies investigate the mechanisms responsible. Our study has identified several key contributors, providing targets for improved graft designs and a starting point for complementary drug therapy to move bioengineered tracheal grafts a step closer to clinical use.

Tracheal disease resulting from cancer, infection, autoimmune conditions, and tracheal injury necessitate approximately 4000 tracheal excisions per year in the United States.^1^^,^^2^ Resection followed by end-to-end anastomosis is the gold standard of tracheal reconstruction. However, with this approach, surgeons must reconstruct tracheas by pulling together the remaining portions of the airway. Even with release maneuvers that relieve tension at the anastomosis, only 4 to 6 cm of the adult trachea can be safely resected, and complications rates are reported to range from 10% to 20%.^3^, ^4^, ^5^, ^6^

One approach to address the length limitation in tracheal reconstruction is to replace the length of trachea resected with a graft. Although there have been rare and one-off attempts to replace the trachea using tissue flaps, autografts and allografts, and synthetic materials, these have met with only limited success,^7^, ^8^, ^9^, ^10^ substantiating the need for an alternate, reliable, and ideally off-the-shelf solution.

Despite early failures due to the premature clinical use of untested tissue-engineered grafts made with decellularized cadaveric tracheas,^11^^,^^12^ engineered grafts still show promise in animal models. Our group has extensive experience using the pig model to study the implementation of a wide range of composite-engineered tracheal graft designs.^13^, ^14^, ^15^ In general, these engineered tracheal grafts have consisted of a decellularized extracellular matrix (ECM) supported by a 3-dimensional-printed polycaprolactone scaffold. It is important to note that a composite graft structure is not further supported by an intraluminal tracheal stent or *t* tube. Circumferential graft coverage ranged from 50% to 100%, spanning the length of 4 to 6 tracheal rings. Smaller circumferential coverage was associated both with longer animal survival time—some animals survived until the study end point at 3 months—and the most extensive luminal epithelialization. More extensive granulation tissue was seen in grafts with greater circumferential coverage. The preclinical assessments of our designs as well as many other groups' designs of engineered tracheal grafts demonstrate the feasibility of creating grafts that accommodate the required mechanics of a replacement airway but show that problems with tissue integration and healing still exist.^13^, ^14^, ^15^, ^16^, ^17^, ^18^, ^19^, ^20^ Most notably, significant obstructing stenosis, due to the development of perianastomotic hypertrophic granulation tissue, remains a persistent problem.

A wide range of cellular and molecular programs and pathways are involved in the complex process of normal wound healing. Inflammatory cytokines and immune cells are involved at the beginning of the wound healing process to attract and differentiate various types of immune cells such as macrophages at the wound site to clean the wound and initiate the healing process. The inflammatory process transitions to a regenerative phase that is characterized by increased cell proliferation and ECM deposition that generates new tissue to fill the wound area. Throughout these phases, growth factors and growth factor signaling pathways are also involved. These drive migration and differentiation of many types of cells, including those of mesenchymal and epithelial lineages to recreate the ECM organization and cellular distribution of native tissue. With such complex and interconnected mechanisms at work to accomplish normal healing, it is yet unclear how perturbations in these processes may derail normal progression and which molecular mechanisms dominate in the formation of nonresolving hypertrophic granulation tissue.

Given that abnormal granulation tissue remains among the central issues with bioengineered tracheal grafts and that the molecular mechanisms driving normal and abnormal healing in the trachea are poorly understood, our goal was to study the gene expression in tracheal granulation tissue from our previous animal models of orthotopic bioengineered tracheal graft replacement to elucidate the molecular drivers.

## Methods

### Animal Samples

All samples were obtained from previous experiments of orthotopic implantation of bioengineered tracheal grafts. Experiments followed the Guide for the Care and Use of Laboratory Animals of the National Institutes of Health and the protocol was approved by the Institutional Animal Care and Use Committee at Mount Sinai Health Systems (May 1, 2015). Surgeries were performed under anesthesia and all efforts were made to minimize pain and suffering. Animals were put to death based on veterinary recommendation when they displayed significant respiratory distress; otherwise, animals were put to death at the study end point of 3 months. After death, the trachea was removed en bloc and fixed in formalin. After fixation, the trachea specimens were trimmed and sections of the anastomoses were embedded in paraffin blocks for further histological sectioning and analysis.

### Degree of Stenosis

Full cross-sections of the trachea taken from the anastomosis region were stained with hematoxylin and eosin. The entire slide was imaged on a Vectra automated slide imaging system with an Olympus microscope at 4× magnification and images were imported into ImageJ (National Institutes of Health) for analysis. The area of the lumen was measured using the freehand selection tool to outline the inner surface of the trachea. An ellipsoid was fitted within the trachea lumen using the cartilage rings as anatomic reference points to approximate the nonstenotic area of the lumen. Percent stenosis was calculated from the difference in area of these 2 measurements.

### Gene Expression

RNA was isolated from slices of formalin-fixed paraffin embedded tissue. Four to 6 10-μm slices were processed using the Qiagen RNeasy FFPE kit according to manufacturer's instructions, with an extended (18 hour) incubation in proteinase K. Genomic DNA contamination was eliminated during RNA isolation by DNase I treatment. The quality of isolated RNA was measured by NanoDrop (Thermo Scientific) and quantified by Qubit (Thermo Scientific) using the RNA broad range kit, then the isolated RNA was stored at −80 °C until use. Copy DNA was synthesized from 5 μg RNA using the SuperScript VILO cDNA Synthesis Kit (ThermoFisher) according to manufacturer's instructions, with an extended 42 °C incubation period (120 minutes) then stored at −20 °C until used. An array of predesigned and validated primers (Qiagen) was used to probe for ∼200 genes related to wound healing. Quantitative polymerase chain reaction was run on a QuantStudio 7 real-time polymerase chain reaction system (Applied Biosystems) with SYBR green chemistry and standard temperature cycling to 40 cycles. Differential gene expression was computed with the ΔΔC_t_ relative expression method using B2M as reference gene. Change in expression was expressed using normal tissue as baseline control.

### Immunohistochemistry

Immunohistochemical staining of proliferating cells and transforming growth factor beta (TGF-β) receptor 1 was performed. For single antigen detection, after heat-mediated antigen retrieval in citrate buffer (pH 6.0), sections were incubated with primary antibodies against either TGFBR1 or proliferating cell nuclear antigen overnight at 4 °C. A biotinylated antimouse and antirabbit secondary antibody was applied and developed with a 3,3′-diaminobenzidine chromogen kit then counterstained with hematoxylin.

### Statistical Analysis

The statistical significance was assessed using 1-way analysis of variance with post hoc Dunnett's test used to compare each stenotic group to the no-stenosis group.

## Results

**Figure 1:**
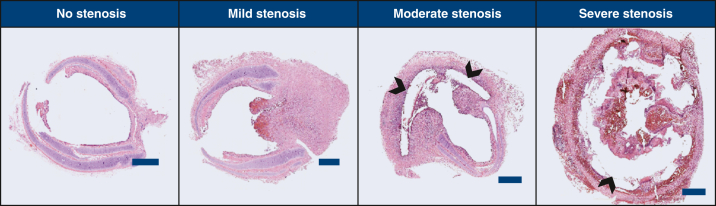
Hematoxylin and eosin-stained histological sections taken from the anastomotic region. Representative images for each extent. Scale bar = 300 μm. *Arrows* indicate void spaces due to the dissolution of the 3-dimensional-printed plastic of the engineered grafts, which was solubilized during the embedding process.

**Figure 2:**
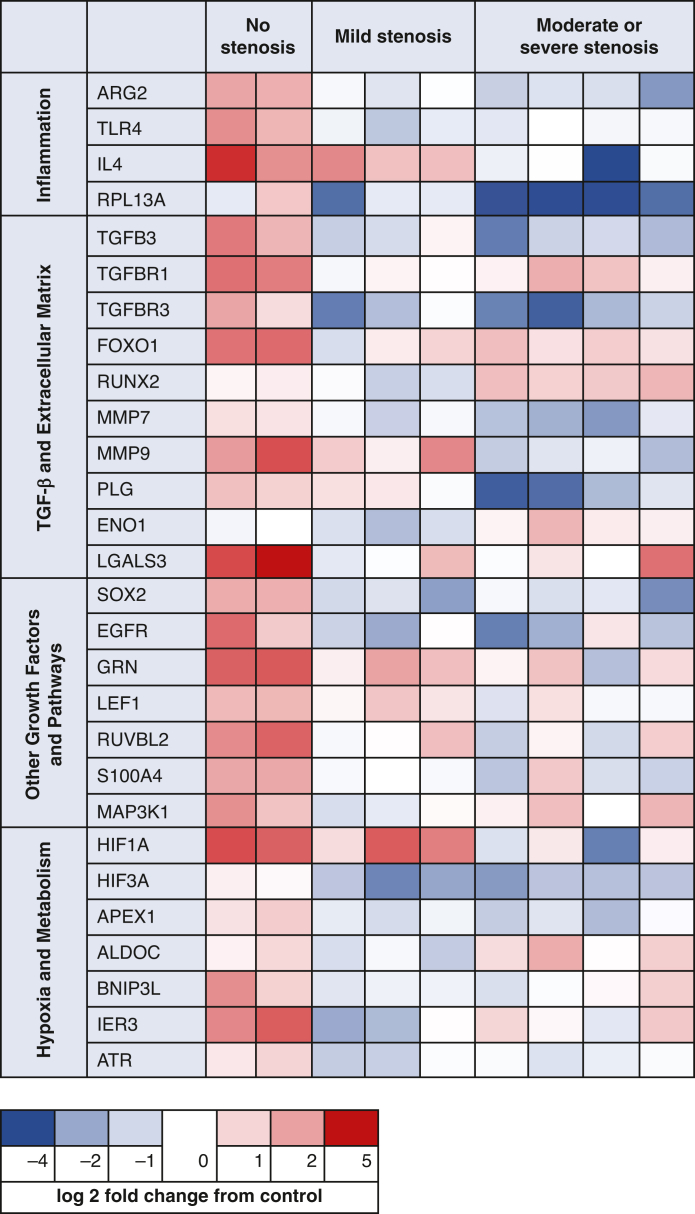
Significantly differentially expressed genes between groups of mild, moderate, severe, and no stenosis (analysis of variance *P* value < .05). Values are normalized by housekeeping gene B2M then by healthy tissue and presented as change from healthy control.

Specimens from 10 animals were used in this study, including 1 animal with no implant that served as control. Test animals were grouped by extent of stenosis (Table 1): no stenosis (<5%), mild stenosis (25%-50%), moderate stenosis (70%-90%), and severe stenosis (>90%). Representative cross-sections for each group are shown in Figure 1. Figure 2 organizes the differentially expressed genes into functional groups and shows the expression levels by heatmap. Table 2 summarizes the change in expression levels and functions of each differentially expressed gene.

**Table 1:** Degree of stenosis∗

Degree of stenosis	Area reduction (%)	No. of animals
No stenosis	3.9 ± 0.3	2
Mild stenosis	39.6 ± 3.7	3
Moderate stenosis	75.1 ± 0.3	2
Severe stenosis	93.2 ± 0.5	2

Percent area reduction values are expressed as mean ± SEM.

∗ To elucidate differences in stenotic versus nonstenotic tracheal wound healing, the expression profile of granulation tissue was assessed using pathway-focused arrays with ∼200 genes. These arrays included genes for extracellular matrix molecules, cell adhesion molecules, inflammatory cytokines and chemokines, growth factors, genes associated with angiogenesis, regulation of cell proliferation and apoptosis, and transcription factors from a variety of signaling pathways. Of the tested genes, 28 were found to be expressed at a significantly different level.

**Table 2:** Significantly differentially expressed genes compared with the no-stenosis group∗

Biological processes involved in	Gene	Change in expression from no stenosis		Function
		Mild	Moderate + severe	
Inflammation	ARG2	↓	↓	Indicative of M2 macrophage^21^
	TLR4	↓	↓	Indicative of M1 and M2 macrophage, modulates inflammation
	IL4		↓	Promotes M2 macrophage, modulates inflammation
	RPL13 A		↓	Indicative of M2 macrophage, modulates inflammation
TGF-β and extracellular matrix	TGFB3	↓	↓	Growth factor, associated with scarless wounds
	TGFBR1	↓	↓	TGF receptor, initiates signaling
	TGFBR3	↓	↓	Matrix component, modulates TGF signaling
	FOXO1	↓	↓	Transcription factor downstream of P13 K/AKT pathway, alternative pathway of TGF-signaling
	RUNX2	↓	↑	TGF-inducible by TGF-β1
	MMP7	↓	↓	Matrix degradation
	MMP9		↓	Matrix degradation
	PLG		↓	Matrix degradation
	ENO1	↓		Plasminogen activator, hypoxia tolerance
	LGALS3	↓	↓	Mediates cell-matrix interaction, promotes epithelial cell migration and differentiation, indicative of M2 macrophage
Other growth factors and pathways	SOX2	↓	↓	Airway-specific transcription factor, promotes angiogenesis^22^
	EGFR	↓	↓	Receptor for epithelial growth factor
	GRN	↓	↓	Acts as growth factor, promotes proliferation of epithelial and endothelial cells and fibroblasts, promotes angiogenesis
	LEF1		↓	Transcription factor in Wnt pathway, expressed by embryonic fibroblasts
	RUVBL2	↓	↓	Modulates LEF1, essential for cilia development and motility
	S100A4	↓	↓	Modulates calcium ion signaling, promotes cell migration
	MAP3K1	↓		Part of NF-kB signaling cascade
Hypoxia and metabolism	HIF3A	↓	↓	Modulates hypoxia-induced genes
	HIF1A		↓	Hypoxic adaptation, promotes angiogenesis
	APEX1	↓	↓	Protects from hypoxic apoptosis
	ALDOC	↓		Epithelial differentiation, glycolysis
	BNIP3L	↓	↓	Induces apoptosis
	IER3	↓	↓	Protects from hypoxic apoptosis
	ATR	↓	↓	Inhibits proliferation

*M2*, Anti-inflammatory macrophages; *M1*, proinflammatory macrophages; *TGF*, transforming growth factor; *P13K/AKT*, phosphoinositide-3-kinase–protein kinase B/Akt; *NF-kB*, nuclear factor kappa B.

∗ Direction indicated by arrow (Dunnett's post-hoc test; *P* < .10). Grouped and annotated by function.

### Inflammatory Pathway and Macrophages

In general, genes associated with regulating inflammatory effects were expressed at a lower level in the stenotic groups compared with the no-stenosis group. ARG2 and TLR4 had lower expression in both stenotic groups compared with no stenosis. In the combined moderate and severe group, both IL4 and RPL13 A expression were lower than no stenosis.

### TGF-β Pathway and ECM Genes

TGFB3 had lower expression in both stenotic groups compared with no stenosis, whereas no difference in expression levels was detected for the other TGFB isoforms. Two of the TGF-β receptors, TGFBR1 and TGFBR3, had lower expression levels in both stenotic groups compared with the no-stenosis group. The transcription factor FOXO1 was also expressed at lower levels in both stenotic groups compared with no stenosis. Compared with the no-stenosis group, the TGF-β1-inducible RUNX2 was expressed at a higher level in the combined moderate and severe group, but lower in the mild group. Immunohistochemistry staining showed increased expression of TGFB2 in the samples with mild and moderate/severe stenosis, with diffuse staining throughout both the epithelium and connective tissue. In comparison, healthy samples or those with no stenosis showed sparse TGFB2 expression.

The matrix-degrading enzymes, MMP7, MMP9, and PLG, had lower expression in the moderate and severe stenosis group compared with the no-stenosis group. MMP7 also had lower expression in the mild stenosis group. The plasminogen activator, ENO1, also had lower expression in the mild stenosis group. For the most part, no detectable differences were observed in gene expression of cell receptors to mechanical stimuli; however, LGALS3 was lower in both stenotic groups compared with no stenosis.

### Other Growth Factors and Growth Factor Pathways

In addition to TGF-β, other growth factor pathways that influence cell proliferation and matrix deposition also had significantly different expression levels from the no-stenosis group. Expression of SOX2, EGFR, and GRN were all expressed at a lower level in the stenotic groups compared with no stenosis. LEF1 was expressed at a lower level in the moderate and severe stenosis groups, whereas RUVBL2 and S100A4 were expressed at a lower level in both stenotic groups compared with no stenosis. Finally, MAP3K1 was expressed at a lower level in the mild stenosis group compared with no stenosis.

Immunohistochemistry staining showed increased expression of proliferating cell nuclear antigen with diffuse staining throughout both the epithelium and connective tissue in the samples with mild or moderate-severe stenosis. In comparison, healthy samples or those with no stenosis showed sparse proliferating cell nuclear antigen expression.

### Hypoxia-induced, Metabolism-related, and Apoptosis Genes

**Figure 3:**
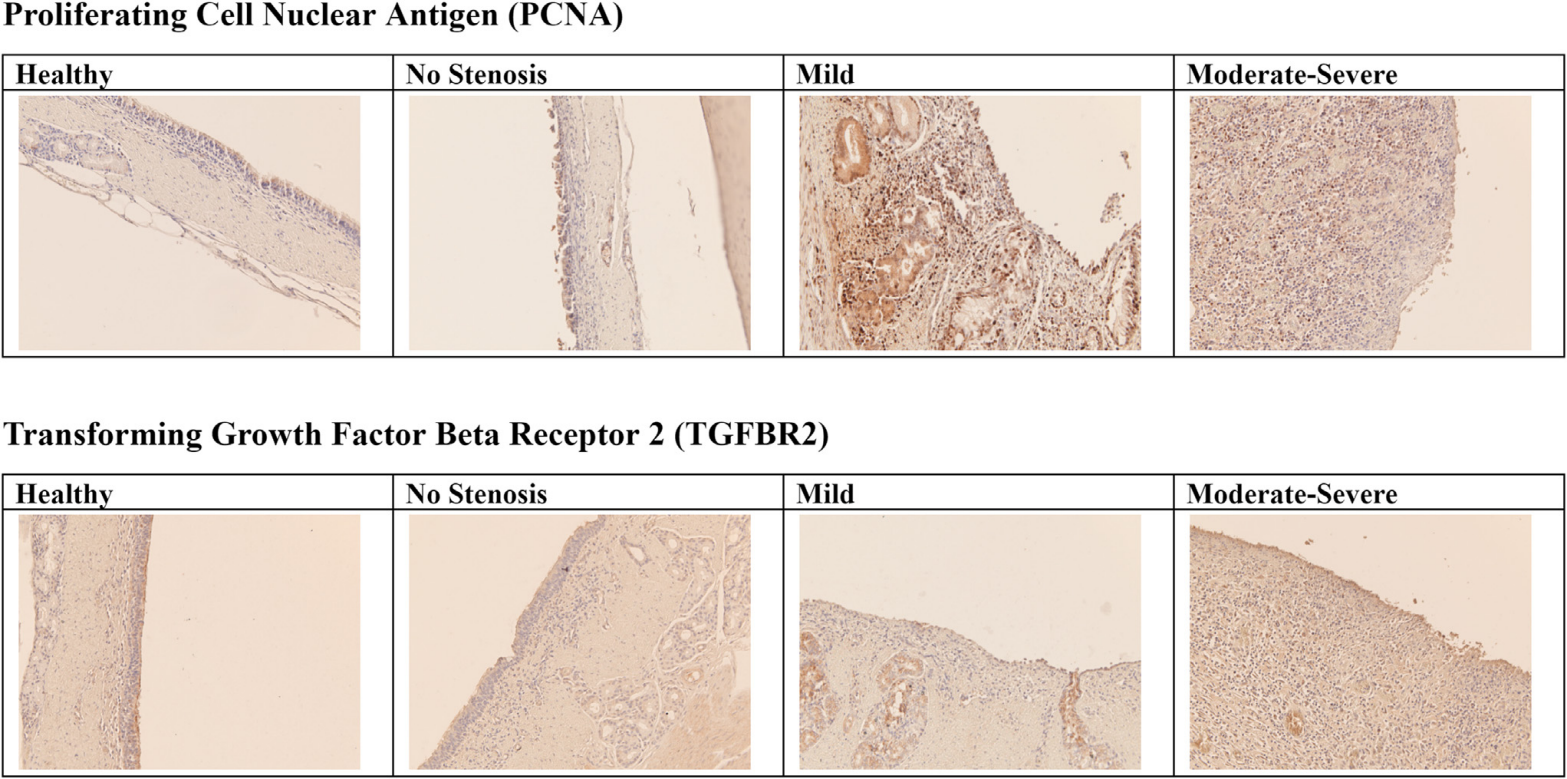
Immunohistochemistry staining for proliferating cell nuclear antigen and TGFBR2. Cell proliferation is indicated by the presence of proliferating cell nuclear antigen; that is, brown nuclei. The likelihood of transforming grown factor β2 signaling is indicated by the presence of TGFBR2 receptor in the cell membrane, cytoplasm, or nuclei.

HIF3A was found to have significantly lower expression in both mild and moderate stenosis compared with no stenosis, whereas HIF1A was only expressed at a lower level in moderate and severe stenosis. The hypoxia-inducible genes APEX1, BNIP3L, IER3, and ATR all had lower expression in moderate and severe stenosis, and these genes plus ALDOC were also expressed at a lower level in mild stenosis (Figure 3).

## Discussion

Hypertrophic perianastomotic granulation tissue is a common failure mode during the in vivo assessment of tissue-engineered tracheal grafts and will be a significant obstacle in the translation of these grafts to clinical use. Although some degree of granulation tissue is expected at the interface of graft and native tissue, the hypertrophic nature of the tissue causes significant airway stenosis leading to morbidity and graft failure.

Our study assessed changes in gene expression from tissue specimens taken from the graft-native interface of orthotopically implanted bioengineered tracheal grafts to identify possible mechanisms responsible for the development and persistence of granulation tissue causing stenosis. This was accomplished using predesigned pathway-focused primer arrays for 200 genes related to wound healing and tissue specimens from 9 animals who had undergone bioengineered trachea implantation plus healthy native tissue from 1 animal to be used as baseline control. Gene expression results were normalized to the healthy native tissue and comparisons of expression levels were made from mild or moderate and severe stenosis to the group of animals with no stenosis to identify differential expression in hypertrophic granulation tissue compared with healing with no stenosis.

The normal healing process is regulated by a multitude of finely balanced and well-timed signals as well as multiple cell types. The abnormal granulation tissue we observed in our implant studies carried many of the typical features of abnormal wounds. The tissue we observed was characterized by high-density fibroblasts in a collagenous matrix with an immature capillary bed. In most cases, the granulation tissue lacked epithelium; however, in cases where it was present, it had lost the pseudostratified columnar structure of functional airway epithelium and was instead metaplastic.

Airway epithelial cells, macrophages, endothelial cells, and fibroblasts are the major cell populations involved in the wound healing process of the trachea. It is clear from our results that there is a complex network of inter- and intracellular signaling involving these cell types that governs both nonstenotic and stenotic healing in response to graft implantation. Contributing to the abnormal healing of the stenotic tracheas includes prolonged inflammation, TGF-β1–induced signaling, a lack of re-epithelialization, and a poor response to hypoxic stress.

### Macrophages and the Inflammatory Response

Proinflammatory (M1) macrophages, are the initial immune responders to injury or biomaterial implantation. M1 macrophages secrete proinflammatory mediators to protect against infection and to initiate tissue degradation that stimulates wound-edge cells to begin migrating and proliferating. Persistent M1 macrophages lead to delayed wound healing by providing a sustained source of proinflammatory cytokines.^23^^,^^24^ In normal healing, M2 macrophages dominate the later phases of wound healing and secrete anti-inflammatory mediators, including the genes IL4 and RPL13 A to curtail the M1-induced inflammation. Timely transition from the M1 to M2 phenotype is necessary for adequate wound healing and tissue repair.^23^^,^^25^ Compared with the no-stenosis group, the stenotic groups appeared to lack these anti-inflammatory M2 macrophages contributing to a sustained inflammatory setting. Although we did not detect differences in inflammatory cytokine gene expression compared with the no-stenosis group, M2 macrophages are capable of modulating inflammation through translational silencing, allowing for a reduction in inflammation even without a corresponding reduction in inflammatory gene expression. Hypoxia has also been shown to contribute to the differentiation of macrophages to an M2 phenotype; thus, the adaptation to oxidative stress we observed in the no-stenosis group helps establish an anti-inflammatory environment that promotes wound healing.

### Epithelial Cells, Fibroblasts, and Proliferation

Re-epithelialization of the wound surface is accomplished by migration and proliferation of wound-edge epithelial cells. SOX2 is a transcription factor highly expressed in airway tissue that is required for the differentiation of nonciliated, goblet, and ciliated cells.^26^ SOX2 has also been identified as a uniquely expressed gene that primes oral wounds for rapid resolution through the expression of EGFR ligands. Therefore, in the airway, SOX2-expressing cells may be primed for quick proliferation to re-epithelialize the wound surface and quick differentiation of the newly produced epithelium into functional airway epithelium. In our stenosis tissue samples, the lack of gene expression for epithelial-specific growth factors and differentiating factors aligned with our observed lack of epithelium in histological sections. Delayed epithelialization is a vicious cycle that allows further damage to unprotected wound tissue perpetuating the wounded condition. Intercellular communication between epithelial cells and fibroblasts is important to mediating wound tissue and the secreted signaling molecules by these cells change based on the cellular environment. Proliferation or senescence can therefore be induced by the continuity of the epithelial layer. Delayed epithelialization is associated with unusual proliferation of fibroblasts and the deposition of excess ECM constituents at the wound site resulting in granulation tissue hypertrophy that causes stenosis.

Signaling in the TGF-β pathway can be initiated by any of the 3 isoforms of TGF-β growth factor; however, different isoforms of TGF-β have been associated with different regenerative potentials, although the specific mechanisms remain unknown. Specifically, the TGF-β3 isoform has been associated with scarless and rapid wound healing and we observed a lower expression of the TGFB3 gene in the stenotic groups. Although TGF-β signaling can increase proliferation and fibrogenesis in fibroblasts and myofibroblasts that are associated with wound closure, excessive proliferation and matrix deposition can occur without modulating factors such as TGFBR3, which controls levels of available growth factor.^27^, ^28^, ^29^, ^30^, ^31^, ^32^ The pleiotropic effects of TGF-β signaling can also be explained by the variety of canonical and noncanonical intracellular signaling pathways that are governed by the cellular environment, including the ratio of available TGF-β isoforms. Although we were unable to detect differences in expression of TGFB1 or 2, the upregulation of RUNX2 suggests that TGF-β signaling in moderate and severe stenosis was initiated by TGF-β1.

### Cell–Matrix Interactions and Matrix Remodeling

Our results indicate a difference in the composition of the ECM between wounds without stenosis and wounds with stenosis. Cells sense and interact with their environment through a variety of receptors. Direct cell–cell contact inhibits spreading and induces senescence of epithelium, whereas loss of contact promotes migration and proliferation. Components of the ECM influence cell behaviors through physiochemical cues. Cells respond not only to the constituents of the ECM, but also to the mechanical signals transduced through the structure. Remodeling of the ECM builds overall tissue strength by replacing the rapidly secreted collagen 3 with much stronger collagen 1 and changes the magnitude of tissue deformation in response to the same degree of force. This type of remodeling is accomplished by a fine balance between very selective proteases such as matrix metalloproteinase and their inhibitors. ECM components also serve as growth factor reservoirs, sequestering excess growth factors to modulate their availability to influence tissue growth. On the other hand, the initial matrix degradation in response to inflammation immediately following tissue injury is accomplished with different and often more broadly targeted proteases that less discriminately degrade tissue structure to allow infiltration of the many cell types needed for wound healing. Prolonged exposure to inflammatory matrix degradation not only weakens the tissue, but also results in sustained release of sequestered growth factors and cytokines and vastly changes the ECM composition, resulting in an abnormal cell response.

## Conclusions

In this work, the differences in gene expression of stenotic and nonstenotic wound tissue in response to trachea graft implantation were described. Our results demonstrate a difference in cell types present and pathways active between stenotic and nonstenotic wounds. A lack of M2-polarized macrophages and epithelial cells along with altered TGF-β signaling via different isoforms were all implicated in the formation of stenotic wounds by the decreased expression of ARG2, IL4, RPL13 A, TGFBR3, and EGFR and increased expression of RUNX2 compared with nonstenotic tissue. These findings represent a significant step for tracheal tissue engineering because wound healing after graft implantation has not been studied to any depth and is an issue that will continue to be a major obstacle in clinical translation. Knowing which genes and molecular pathways are involved in stenotic versus nonstenotic healing is a useful start for deeper investigations of the underlying mechanisms and identification of potential treatments.

### Conflict of Interest Statement

Dr Bhora has consulting relationships with the following that are unrelated to this study: AstraZeneca, Ambu, Genentech, Biodesix, Johnson & Johnson/Ethicon, Boston Scientific, and Medtronic. All other authors reported no conflicts of interest.

The *Journal* policy requires editors and reviewers to disclose conflicts of interest and to decline handling manuscripts for which they may have a conflict of interest. The editors and reviewers of this article have no conflicts of interest.
